# Differentially private genome data dissemination through top-down specialization

**DOI:** 10.1186/1472-6947-14-S1-S2

**Published:** 2014-12-08

**Authors:** Shuang Wang, Noman Mohammed, Rui Chen

**Affiliations:** 1 Division of Biomedical Informatics, University of California-San Diego, San Diego, CA, 92093, USA; 2 Department of Computer Science, University of Manitoba, Manitoba, Winnipeg, MB R3T 2N2, Canada; 3 Department of Computer Science, Hong Kong Baptist University, Kowloon Tong, Hong Kong

**Keywords:** Genome-wide association studies (GWAS), differential privacy, data dissemination

## Abstract

Advanced sequencing techniques make large genome data available at an unprecedented speed and reduced cost. Genome data sharing has the potential to facilitate significant medical breakthroughs. However, privacy concerns have impeded efficient genome data sharing. In this paper, we present a novel approach for disseminating genomic data while satisfying differential privacy. The proposed algorithm splits raw genome sequences into blocks, subdivides the blocks in a top-down fashion, and finally adds noise to counts to preserve privacy. The experimental results suggest that the proposed algorithm can retain certain data utility in terms of a high sensitivity.

## Introduction

Recent advances in genome sequencing techniques have the potential to speed up scientific discoveries and enable significant medical breakthroughs. Meanwhile, they also raise important concerns about the privacy of individuals. For example, Homer's attack [1] demonstrated that it is possible to identify a genome-wide association study (GWAS) participant from the allele frequencies of a large number of single-nucleotide polymorphisms (SNPs). Due to these and other potential privacy risks, NIH has forbidden public access to most aggregate research results to protect privacy. Wang et al. [2] showed an even higher risk that individuals could be actually identified from a relatively small set of statistics such as those routinely published in GWAS papers. There are many other attacks revealed recently [3-5], which could result in harm to the privacy of individuals. It is a big challenge to promote privacy-preserving data sharing for genomic research. In the United States, the Health Insurance Portability and Accountability Act (HIPPA) [6] establishes the Privacy Rule to protect health information. The Privacy Rule establishes an operational approach, called Safe Harbor that removes 18 HIPAA-specified identifiers to achieve some degree of "de-identification". Since genome data are biometrics, it would be natural to remove these data from "de-identified" data sets. However, there is no explicit clarification of de-identified genomic data by the Institute of Medicine (IOM) or HIPAA regulations. There have been long and vigorous debates [7,8] about the current privacy rules for Human Genomic Studies (HGS). Some researchers contend that existing privacy rules are not adequate for the protection of genomic information [2,9], as the technological evolution and the increasing accessibility of data cause the "de-identified" genome data to be re-identifiable. Others complain that privacy regulations impede effective data access and use for research, as genomic data are most useful when presented in high quality, sufficient samples, and associated with an individual's medical history, etc. Recently, the Presidential Commission for the Study of Bioethical Issues published a report about privacy and progress in Whole Genome Sequencing (WGS) [10]. The report concludes that under current privacy rules, genome privacy is not adequately protected and that at the same time genomic researchers and data owners cannot effectively access and share them. To address these limitations, there have been several efforts on developing practical privacy-preserving technology solutions.

## Problem statement

Suppose a data owner has a data table *D*(*A^i^, Asn^p^*) and wants to release an anonymous data table $\hat{D}$ to the public for data analysis. The attributes in *D *are classified into two categories: (1) An *explicit identifier *attribute that explicitly identifies an individual, such as *SSN*, and *Name*. These attributes are removed before releasing the data as per the HIPAA Privacy Rule [11]. (2) A set of SNPs (genomic data), which is denoted by *A^snp^*, for each individual in the data table *D*.

Given a data table *D*, our objective is to generate an anonymized data table $\hat{D}$ such that (1) $\hat{D}$ satisfies *E*-differential privacy, and (2) preserves as much utility as possible for data analysis. Next, we introduce differential privacy and data utility models.

### Privacy protection model

Differential privacy is a recent privacy definition that provides a strong privacy guarantee. It guarantees that an adversary learns nothing more about an individual from the released data set, regardless of whether her record is present or absent in the original data. Informally, a differentially private output is insensitive to any particular record. Therefore, from an individual's point of view, the output is computed as if from a data set that does not contain her record.

**Definition **(*ε-Differential Privacy*) [12] A randomized algorithm *Ag *is differentially private if for all data sets *D *and *D' *whose symmetric difference contains at most one record (i.e., *|D *Δ *D'| ≤ *1), and for all possible anonymized data sets $\hat{D}$,

(1) $$\text{Pr}\left[Ag\left(D\right)=\hat{D}\right]\leq re^{\varepsilon }\times \text{Pr}\left[Ag\left(D^{\prime }\right)=\hat{D}\right]$$

A standard mechanism to achieve differential privacy is to add random noise to the true output of a function. The noise is calibrated according to the *sensitivity *of the function. The sensitivity of a function is the maximum difference of its outputs from two data sets that differ only in one record. This is also known as Laplacian mechanism [12].

### Privacy attack model

The likelihood ratio test [13] provides an upper bound on the power of any method for the detection of an individual in a cohort, using the following formula:

$$\bar{L}=\overset{m}{\underset{j}{\sum }}\left(x_{j}\text{log}\frac{{\hat{p}}_{j}}{p_{j}}+\left(1-x_{j}\right)\text{log}\frac{1-{\hat{p}}_{j}}{1-p_{j}}\right),$$

where *x_j _*is either 0 (i.e., major allele) or 1 (i.e., minor allele), *m *is the number of SNPs, *p_j _*is the allele frequency of SNP *j *in the population and ${\hat{p}}_{j}$ is that in a pool.

### Utility criteria

We use a case-control association test to evaluate the utility of a differentially private data. The test has the following form: $\chi^{2}=\sum_{i}^{r}\sum_{j}^{c}\frac{{\left(O_{i,j}-E_{i,j}\right)}^{2}}{E_{i,j}}$, where *r *is the number of rows, *c *is the number of columns, *O_i,j _*is observed frequencies, and *E_i,j _*is expected frequencies.

**Algorithm 1 **Genomic Data Anonymization

*· ***Input: **Raw data set *D*, privacy budget *ε*, and number of specializations *h*

*· ***Output: **Anonymized genomic data set *D*ˆ

1: Divide the genome data into blocks;

2: Generate the taxonomy tree for each block;

3: Initialize every block in *D *to the topmost value;

4: Initialize *Cut_i _*to include the topmost value;

5: **for ***i *= 1 to *h ***do**

6:    Select *v *∈ ∪*Cut_i _*randomly;

7:    Specialize *v *on *D *and update ∪*Cut_i_*;

8: **end for**

9: **return **each leaf node with noisy count (*C *+ Lap(1*/E*))

## Genomic data anonymization

In this section, we first present our genomic data anonymization algorithm as described in Algorithm 1 and prove that the algorithm is *E*-differentially private. We then analyze the runtime complexity of the algorithm.

### Anonymization algorithm

The proposed algorithm first divides the genomic data into blocks and then generalizes each block. Thus, the algorithm divides the raw data into several equivalence groups, where all the records within a group have the same block values. Finally, the algorithm publishes the noisy counts of the groups. Next we elaborate each line of the algorithm.

**Dividing the raw data (Line 1)**. Algorithm 1 first divides the raw genomic data into multiple blocks. Each block consists of a number of SNPs. For example, the raw genomic data of Table 1 can be divided into 4 blocks as shown in Table 2, where each block consists of two SNPs. These blocks are treated like different attributes and thus enable the proposed algorithm to anonymize high-dimensional genomic data effectively. We denote each block by $A_{i}^{snp}$ and thus $A^{snp}=\cup A_{i}^{snp}$.

**Table 1:** Raw genome data.

ID	Genomic data
1	AG CC CC GG CT GG AA CC
2	AG CC CC GG TT GG AA CC
3	AA CC CC GG TT GG AA CC
4	AG CT CT AG CT AG AG CT
5	GG CT CT AG CC GG AA CC
6	AA CC CC GG TT GG AA CC
7	AG CT CT AG CT AG AG CT
8	AA CC CC GG TT GG AA CC
9	GG CT TT AG CC AG AA CC
10	AG CT CT GG CT AG AA CC

**Table 2:** Genome data partitioned into blocks.

ID	Genomic data			
	**Block 1**	**Block 2**	**Block 3**	**Block 4**
1	AG CC	CC GG	CT GG	AA CC
2	AG CC	CC GG	TT GG	AA CC
3	AA CC	CC GG	TT GG	AA CC
4	AG CT	CT AG	CT AG	AG CT
5	GG CT	CT AG	CC GG	AA CC
6	AA CC	CC GG	TT GG	AA CC
7	AG CT	CT AG	CT AG	AG CT
8	AA CC	CC GG	TT GG	AA CC
9	GG CT	TT AG	CC AG	AA CC
10	AG CT	CT GG	CT AG	AA CC

Note that the sizes of all the blocks do not need to be equal. For example, if there were nine SNPs in Table 1 instead of 8, it would be impossible to have all blocks of size two. In such a case, the last block can be bigger than the other blocks. In principle, each block may have a different size, and the proposed algorithm can handle such a scenario.

We do not use any heuristic to determine the size of each block. Experimental results suggest that six SNPs per block yield good result. However, this number may vary depending on the data set in question. It is an interesting research problem to design a heuristic that can determine the optimal size of each block so as to maximize the data utility for a given data set.

**Figure 1:**
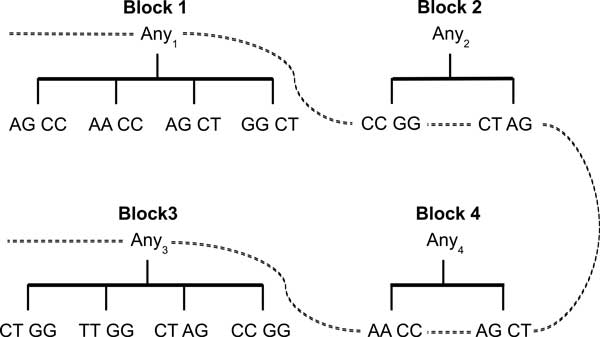
**Taxonomy tree of blocks**.

**Generating the taxonomy tree (Line 2)**. A taxonomy tree of a block $A_{i}^{snp}$ specifies the hierarchy among the values. Figure 1 presents the taxonomy trees of Blocks 1 *− *4 (ignore the dashed curve for now) in Table 2. A *cut *of the taxonomy tree for a block $A_{i}^{snp}$, denoted by *Cut_i_*, contains exactly one value on each root-to-leaf path (more discussion follows).

Ideally, the data owner should provide a taxonomy tree for each block as the knowledge of the taxonomy tree is domain specific. However, if no taxonomy tree is provided, Algorithm 1 can generate it by scanning the data set once for each block. For each unique value that appears in the data set, a leaf node is created from the root node *Any*1. For example, four unique values (i.e., AG CC, AA CC, AG CT, and GG CT) appear in Table 2 for Block 1; therefore, the corresponding taxonomy tree also has four leaves as shown in Figure 1. All the generated taxonomy trees have only two levels (i.e., root and the leaf nodes). However, a data owner can define a multilevel taxonomy tree for each block [14]. Multilevel taxonomy tree provides more flexibility and may preserve more data utility; further investigation is needed to validate the benefit of multilevel taxonomy trees.

**Data anonymization (Lines 3-8)**. Anonymization starts by creating a single root partition by generalizing all values in $\cup A_{i}^{snp}$ to the topmost value in their taxonomy trees (Line 3). The initial *Cuti *contains the topmost value for each block $A_{i}^{snp}$ (Line 4).

**Figure 2:**
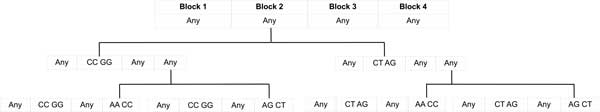
**Tree for partitioning records**.

The specialization starts from the topmost cut and pushes down the cut iteratively by specializing some value in the current cut. The general idea is to anonymize the raw data by a sequence of specializations, starting from the topmost general state as shown in Figure 2. A *specialization*, denoted by *v → child*(*v*), where *child*(*v*) is the set of child values of *v*, replaces the parent value *v *with a child value. The specialization process can be viewed as pushing the "cut" of each taxonomy tree downwards. Figure 1 shows a solution cut indicated by the dashed curve corresponding to the anonymous Table 3.

**Table 3:** Anonymous data (*ε *= 1, *h *= 2).

Genomic data	Noisy Count
Any CC GG Any AA CC	3
Any CC GG Any AG CT	2
Any CT AG Any AA CC	1
Any CT AG Any AG CT	3

At each iteration, Algorithm 1 randomly selects a candidate *v *∈ ∪*Cut_i _*for specialization (Line 6). Candidates can be selected based on their score values, and different heuristics (e.g., information gain) can be used to determine candidates' scores. In future work, we will investigate how to design a scoring function tailored to a specific data utility requirement.

Then, the algorithm specializes *v *and updates ∪*Cut_i _*(Line 7). Algorithm 1 specializes *v *by recursively distributing the records from the parent partition into disjoint child partitions with more specific values based on the taxonomy tree. The algorithm terminates after a given number of specializations.

**Example 1 ***Consider *Table 1*with ε = 1 and h = 2. Initially the algorithm creates one root partition containing all the records that are generalized to *〈*Any*_1_*, Any*_2_*, Any*_3_*, Any*_4_〉. ∪*Cut_i _includes *{*Any*_1_*, Any*_2_*, Any*_3_*, Any*_4_}*. Let the first specialization be Any*_2 _*→ *{*CC GG, CT AG*}*. The algorithm creates two new partitions under the root, as shown in *Figure 2, and *splits data records between them*. ∪*Cut_i _is updated to *{*Any*_1_*, Any*_3_*, Any*_4_}*. Suppose that the next specialization is Any*_4 _*→ *{*AA CC, AG CT *}*, which creates further specialized partitions, as illustrated in *Figure 2.

**Returning the noisy counts (Line 9)**. Finally, Algorithm 1 computes the noisy count of each leaf partition to construct the anonymous data table $\hat{D}$ as shown in Table 3. The number of leaf partitions is at least 2*^h ^*and the exact number depends on the taxonomy tree of the blocks.

Publishing the true counts of each partition violates differential privacy; therefore, a random variable Lap(Δ*f/ε*) is added to the true count of each leaf partition, where Δ*f *= 1.

### Privacy analysis

We now analyze the privacy implication of each of the above steps and quantify the information leakage in terms of privacy budget.

**Line 1**. The algorithm divides the raw data into blocks, where the block size is a given constant irrespective of the given data set. Since the block generation process is data independent, this step does not require any privacy budget. However, if a heuristic were used to determine the block size, then a portion of privacy budget should be allocated to satisfy differential privacy.

**Line 2**. We assume that the data owner provides the taxonomy trees. In such a case, this step incurs no privacy leakage and no privacy budget is consumed as the taxonomy trees are generated from public knowledge that is independent of any particular data set.

On the other hand, the alternative approach that we outlined, for a scenario when the taxonomy trees are not provided, needs additional treatment to satisfy differential privacy. It is because, for a different data set $\hat{D}$, a taxonomy tree may have one more or less leaf node. We argue that taxonomy trees represent the domain knowledge, and therefore, should be part of public information.

**Lines 3-8**. The algorithm selects a candidate for specialization randomly (Line 7) and iteratively creates child partitions based on the given taxonomy trees (Line 8). Both operations are independent of the underlining data set (the selection process is random and the partitioning process is fixed due to the given taxonomy trees), and therefore no privacy budget is required for the *h *number of iterations.

**Line 9**. The algorithm adds Laplace noise Lap(1*/ε*) to the true count of each leaf partition and the requisite privacy budget is *ε *due to the *parallel composition property *[15]. The Parallel composition property guarantees that if a sequence of computations are conducted on *disjoint *data sets, then the privacy cost does not accumulate but depends only on the worst guarantee of all the computations. Since the leaf partitions are disjoint (i.e., a record can fall into exactly one leaf partition), the total privacy cost (i.e., the budget required) for this step is *ε*.

In conclusion, Line 1, Line 2, Lines 3-8, and Line 9 use 0, 0, 0, and *ε *privacy budgets, respectively. According to the *sequential composition property *of differential privacy [15], any sequence of computations that each provides differential privacy in isolation also provides differential privacy in sequence. Therefore, Algorithm 1 satisfies *ε*-differential privacy.

### Computational complexity

The proposed algorithm is scalable and the runtime is linear to the size of the data set. This is an important property to achieve in the age of big data. In this section, we present a brief analysis of the computational complexity of Algorithm 1.

**Line 1**. Algorithm 1 generates the blocks from the raw data. This can be done by scanning the data set once. Thus, the runtime of this step is *O*(*|D| × m*), where *|D| *is the number of records and *m *is the number of SNPs.

**Line 2**. In case, algorithm 1 can also generate the taxonomy trees (if not given) by scanning the data set once. This is can be achieved simultaneously with the previous step (Line 1); hence, there is no additional cost for generating taxonomy trees.

**Lines 3-8**. Candidates are selected randomly in each iteration, which requires constant *O*(1) time (Line 6).

To perform a specialization *v → child*(*v*), we need to retrieve *D_v_*, the set of data records generalized to *v*. To facilitate this operation we organize the records in a tree structure as shown in Figure 2. Each leaf partition (node) stores the set of data records having the same generalized block values. This will allow us to calculate the noisy counts in Line 9.

Initially, the tree has only one leaf partition containing all data records, generalized to the topmost value on every block. In each iteration we perform a specialization by refining the leaf partitions and splitting the records among the new child partitions. This operation also requires scanning all the records once per iteration. Thus, the runtime of this step is *O*(*|D| × h*). The value of *h *is constant and usually very small (around 10), and therefore, can be ignored.

**Line 9**. The cost of adding Laplace noise is proportional to the number of leaf nodes, which is at least 2*^h^*. For a small value of *h*, the number of leaf nodes is insignificant with respect to the size of the data set *|D|*. We therefore can ignore the cost of this step. Note that, we can easily determine the true count of a leaf partition as it keeps track of the set of data records it represents.

Hence, the total runtime of the algorithm is *O*(*|D| × m *+ *|D|*) = *O*(*|D| × m*).

## Experimental results

The goal of the proposed framework is to generate differentially private data that can mitigate the attack of likelihood ratio tests, while preserving highly significant SNPs as much as possible. Two data sets (i.e., chr2 and chr10) with 200 participants in case, control and test groups were used in our experiments, which were obtained from the Human Genome Privacy Challenge [16]. Besides, the chr2 and chr10 data sets contain 311 SNPs and 610 SNPs, respectively.

### Experimental results

The number of specializations used in our experiment was 5. SNP data were split evenly into *N/*6 blocks, where *N *is the number of SNP. All the results are based on the average of 100 trials.

Tables 4 and 5 illustrate the results of the proposed method on chr2 and chr10 data sets with privacy budget of 1.0, where power indicates the ratio of identifiable individuals using the likelihood ratio test in the case group. In Tables 4 and 5, cutoff p-value thresholds of 5E-2, 1E-2, 1E-3, 1E-5 were used in our experiment, for which four measurements (accuracy, sensitivity, precision and F1-score) were calculated under each method. The last column corresponds to the number of significant SNPs discovered in the original data without adding noise. We can see that the proposed results showed high sensitivities but low precisions on both data sets, which means our method can correctly preserve most true significant SNPs, but with a large amount of false positive reports.

**Table 4:** Data utility of chr2 data set with privacy budget of 1.0 and power of 0.01.

Cutoff p-value	Accuracy	Sensitivity	Precision	F1-score	# of significant SNPs
5E-02	0.178	1.000	0.079	0.147	22
1E-02	0.211	0.999	0.075	0.140	20
1E-03	0.250	0.948	0.072	0.134	19
1E-05	0.297	1.000	0.060	0.114	14

**Table 5:** Data utility of chr10 data set with privacy budget of 1.0 and power of 0.09.

Cutoff p-value	Accuracy	Sensitivity	Precision	F1-score	# of significant SNPs
5E-02	0.301	0.956	0.092	0.168	45
1E-02	0.317	0.903	0.048	0.091	23
1E-03	0.431	1.000	0.041	0.080	15
1E-05	0.577	1.000	0.030	0.058	8

**Figure 3:**
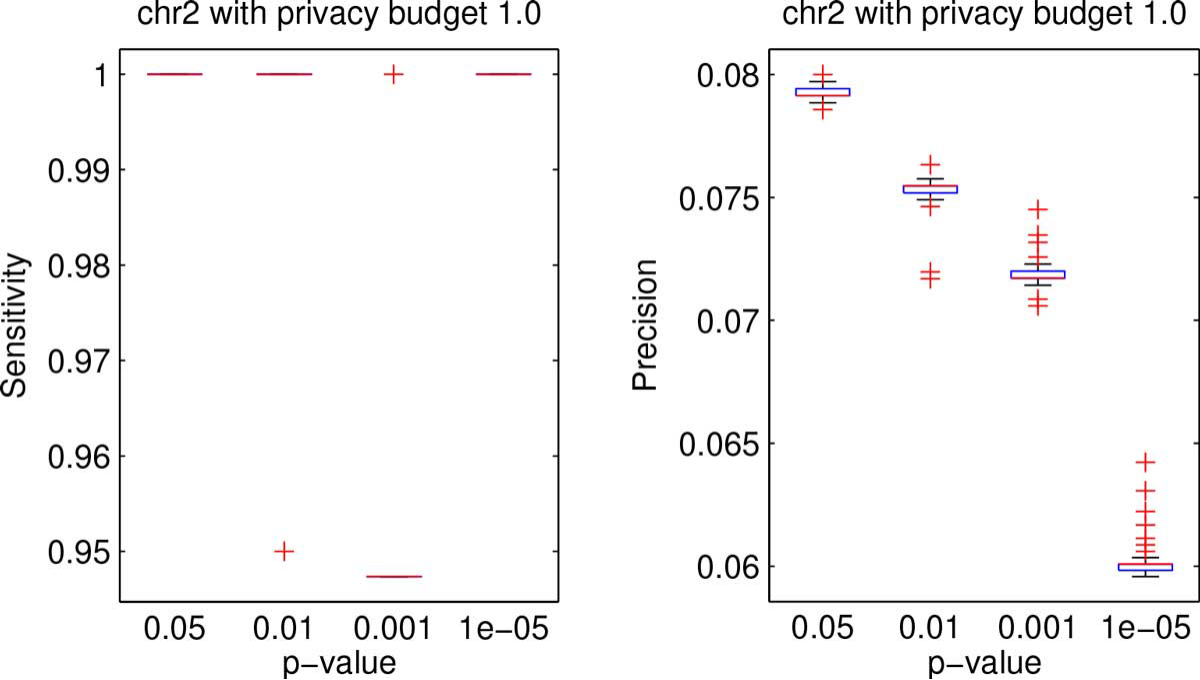
**Boxplots of data utility of chr2 data with different p-values**.

**Figure 4:**
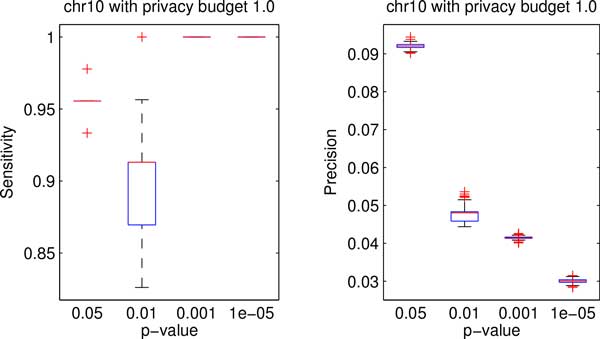
**Boxplots of data utility of chr10 data with different p-values**.

Figures 3 and 4 show the box plots of the data utility in terms of sensitivity and precision for both testing data sets with privacy budget of 1.0 under different cutoff p-values. We can see that the proposed method achieved high sensitivity on both data sets for all cutoff p-values. Moreover, Figures 3 and 4 depict that the precision decreases as the cutoff p-value decreases.

**Figure 5:**
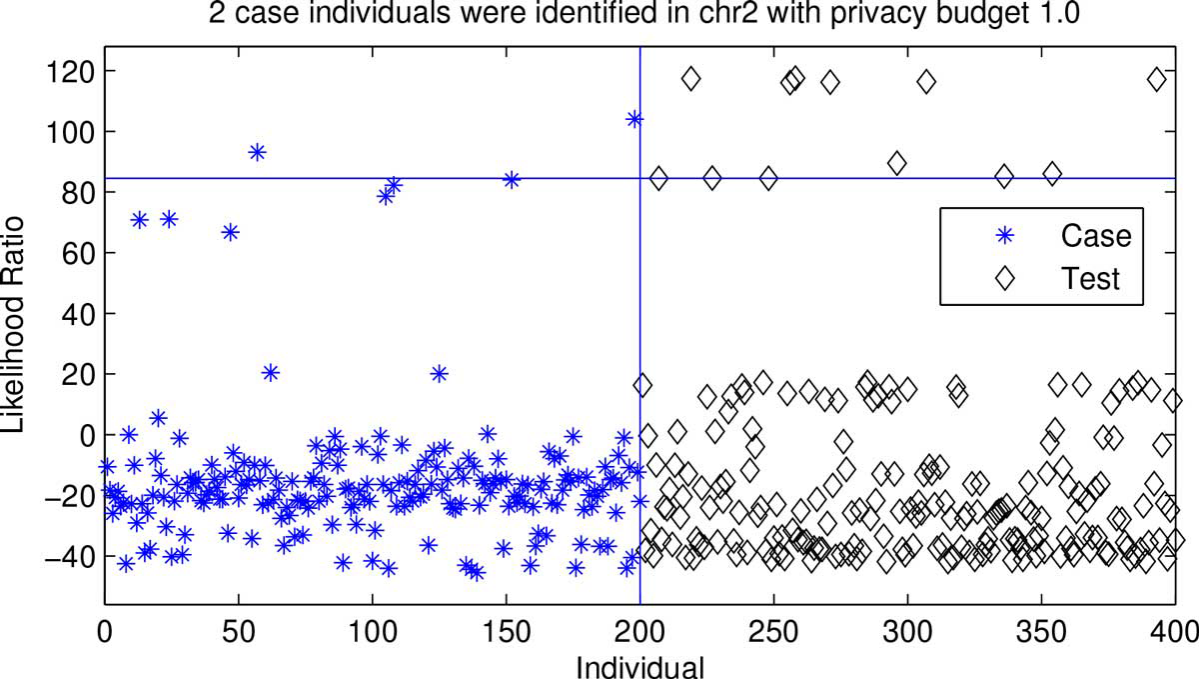
**Privacy risk of chr2 data**. The star and diamond markers represent the test value of a specific individual in the case (left) or test (right) group, respectively. The horizontal line indicates the 0.95 confidence level for identifying case individuals that are estimated based on the test statistic values of test individuals.

**Figure 6:**
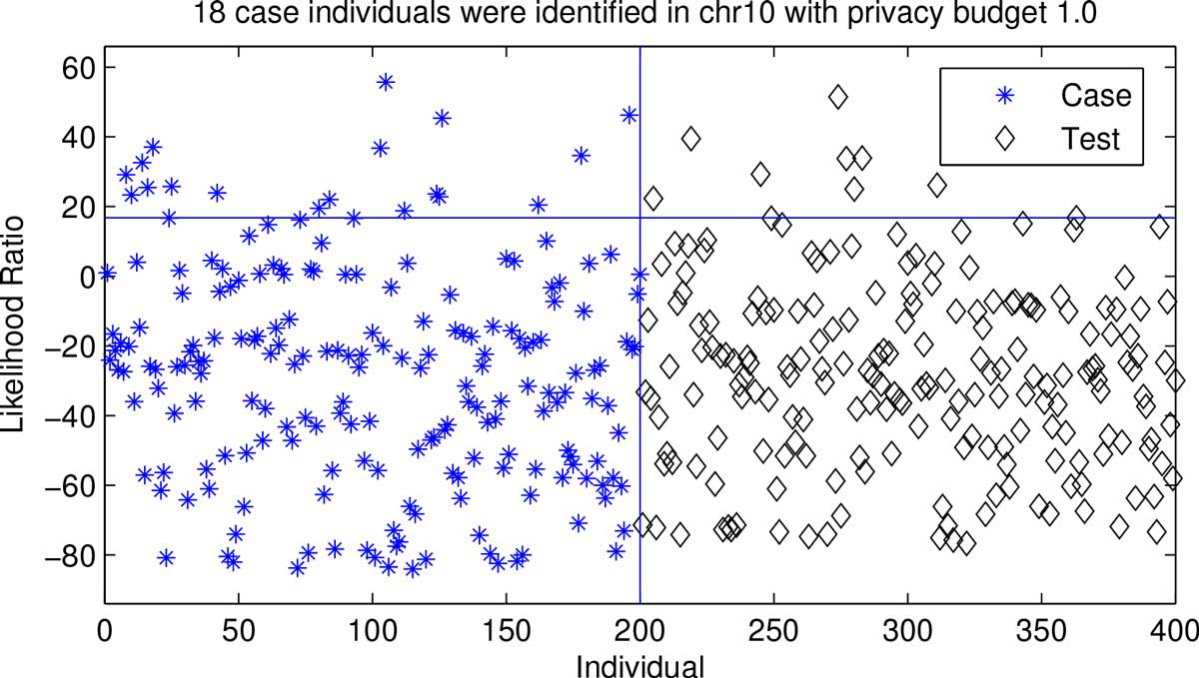
**Privacy risk of chr10 data**. The star and diamond markers represent the test value of a specific individual in the case (left) or test (right) group, respectively. The horizontal line indicates the 0.95 confidence level for identifying case individuals that are estimated based on the test statistic values of test individuals.

Figures 5 and 6 present the test statistics calculated on case, control and test groups (i.e., individual unrelated to both case and control) for both chr2 and chr10 data sets. An individual in the case group can be re-identified with a high confidence if the test statistic obtained from his/her SNP sequence is significantly higher than these of the test group using likelihood ratio test [1]. Figures 5 and 6 depict that 2 and 18 case individuals have higher test statistic values than 95% test individuals (i.e., a 5% false positive rate) in both data sets. The results suggest that the proposed method provides a better privacy protection on a small data set (i.e., chr2 data set) under the same privacy budget.

**Figure 7:**
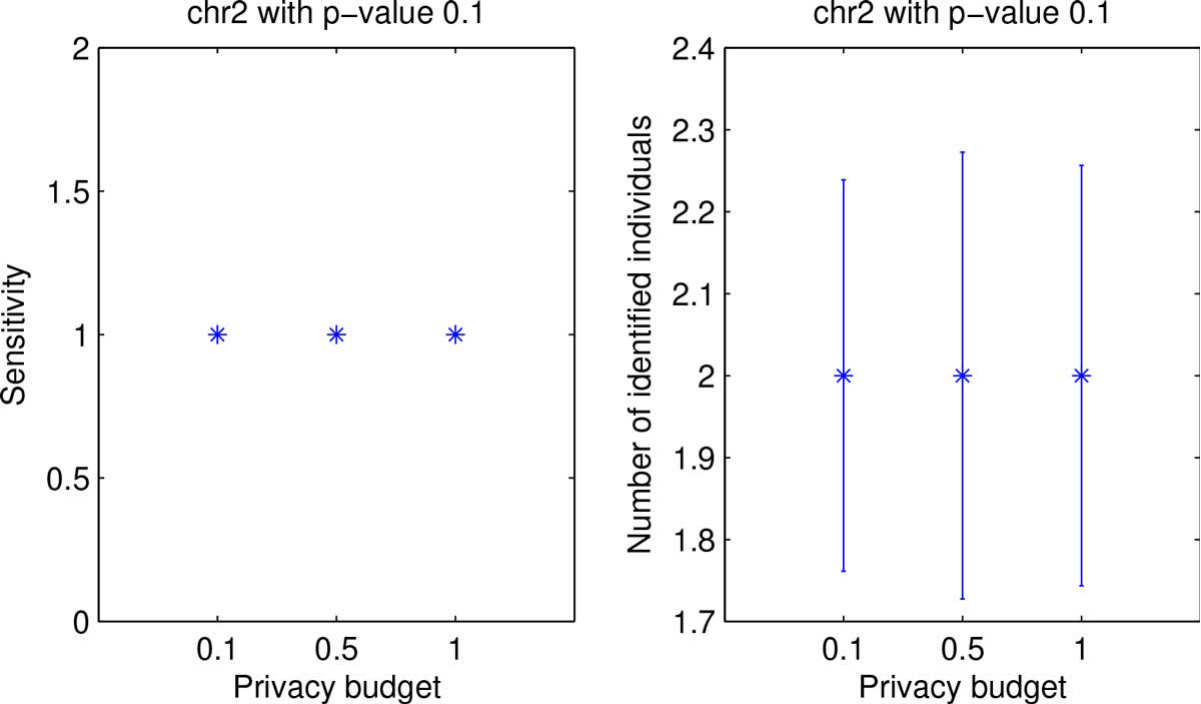
**Comparison of data utility and privacy risk for chr2 data with different privacy budget**.

**Figure 8:**
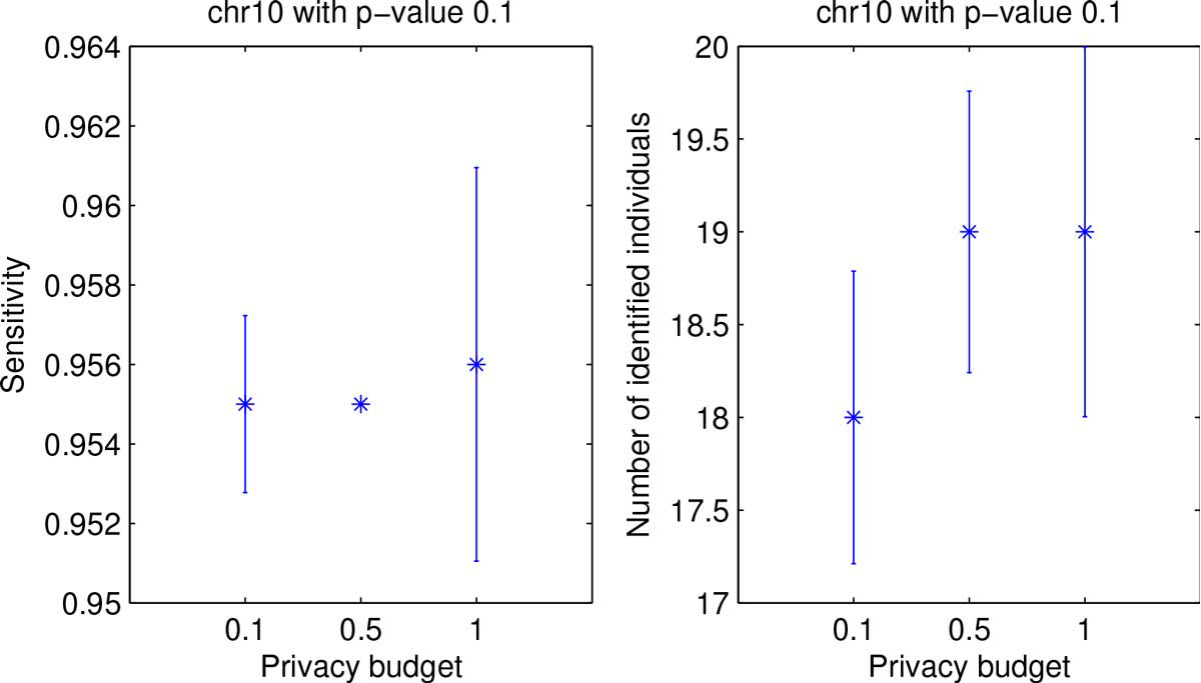
**Comparison of data utility and privacy risk for chr10 data with different privacy budget**.

Finally, Figures 7 and 8 show both utility and privacy risk for chr2 and chr10 data sets. By changing privacy budget from 0.1 to 1, we observw no performance gain of sensitivity nor privacy risk change on chr2 data set, as shown in Figure 7. We also tested the proposed algorithm on a larger data set (i.e., chr10). Figure 8 shows that the proposed algorithm achieves the best sensitivity and the highest number of re-identification risk with privacy budget of 1.0.

## Conclusions

In summary, we developed a novel approach to disseminate genomic data in a privacy-preserving manner. The privacy guarantee is guarded by the rigorous differential privacy model. Our approach uses a top-down structure to split long sequences into segments before adding noise to mask record owners' identity, which demonstrates promising utility with desirable computational complexity. The experimental results suggest that the proposed algorithm can retain data utility with a high sensitivity. The proposed algorithm can also be used to protect heterogeneous data, such as records consisting of both medical and genomic data. The proposal framework also has limitations. For example, the precision performance of the proposed framework is relatively poor. Further improvement is possible by refining the heuristic for splitting sequences and by introducing a scoring function in the data specialization process.

## Competing interests

The authors declare that they have no competing interests.

## Authors' contributions

SW and NM drafted the majority of the manuscript, SW conducted the experiments. RC guided the experimental design.

## Declarations

Publication of this article has been funded by iDASH (U54HL108460), NHGRI (K99HG008175), NLM (R00LM011392,R21LM012060),CTSA(UL1TR000100), NCBC-linked grant (R01HG007078) and NSERC postdoctoral fellowship.

This article has been published as part of *BMC Medical Informatics and Decision Making *Volume 14 Supplement 1, 2014: Critical Assessment of Data Privacy and Protection (CADPP). The full contents of the supplement are available online at http://www.biomedcentral.com/bmcmedinformdecismak/supplements/14/S1.
